# Assessment of a Multiplex LAMP Assay (Eazyplex^®^ CSF Direct M) for Rapid Molecular Diagnosis of Bacterial Meningitis: Accuracy and Pitfalls

**DOI:** 10.3390/microorganisms9091859

**Published:** 2021-09-01

**Authors:** Anne-Gaëlle Leroy, Elise Persyn, Sophie-Anne Gibaud, Lise Crémet, Paul Le Turnier, Myriam Benhamida, Elise Launay, Aurélie Guillouzouic, Pascale Bémer, Stéphane Corvec, on behalf of the Western French Study Group on Early Bacterial Meningitis

**Affiliations:** 1 Department of Microbiology, University Hospital of Nantes, 44093 Nantes, France; elise.persyn@chu-nantes.fr (E.P.); sophieanne.gibaud@chu-nantes.fr (S.-A.G.); lise.cremet@chu-nantes.fr (L.C.); aurelie.guillouzouic@chu-nantes.fr (A.G.); pascale.bemer@chu-nantes.fr (P.B.); stephane.corvec@chu-nantes.fr (S.C.); 2 EA 3826 “Thérapeutiques Cliniques et Expérimentales des Infections”, Institut de Recherche en Santé 2-Nantes Biotech, Medical University of Nantes, 44000 Nantes, France; 3 Department of Infectious Diseases, University Hospital of Nantes, 44093 Nantes, France; paul.leturnier@chu-nantes.fr; 4 INSERM CIC 1413, University Hospital of Nantes, 44093 Nantes, France; 5 Clinique Médicale Pédiatrique, University Hospital of Nantes, 44093 Nantes, France; myriam.benhamida@chu-nantes.fr (M.B.); elise.launay@chu-nantes.fr (E.L.); 6 CRCINA, INSERM U1232, University Hospital of Nantes, 44093 Nantes, France

**Keywords:** bacterial meningitis, rapid diagnosis, Eazyplex^®^ CSF direct M panel, Loop-Mediated Isothermal Amplification (LAMP), performance, amplification curves analysis

## Abstract

*Background:* Automated molecular panels are attractive tools for improving early meningitis diagnosis. This study assessed the Eazyplex^®^ CSF direct M panel (EP), a multiplex real-time Loop-Mediated Isothermal Amplification assay. *Methods:* From December 2016 to December 2019, cerebrospinal fluid (CSF) samples were routinely tested with the EP V1.0. CSF parameters and microbiological and clinical data were retrospectively collected. *Results:* Out of 230 CSF samples, the EP yielded positive, negative, and invalid results for 32 (13.9%) (16 *N. meningitidis*, nine *S. pneumoniae*, two *S. agalactiae*, two *E. coli*, two *H. influenzae*, one *L. monocytogenes*), 182 (79.1%), and 16 (7%) samples, respectively. Among the positive samples, 14 (44%) remained negative in culture (antibiotic therapy before lumbar puncture (*n* = 11), meningococcal meningitis (*n* = 3)). High CSF protein concentrations and cellularity were associated with LAMP inhibition, counteracted by centrifugation. The automated software yielded 13 false positive and five false negative results. Amplification curve analysis was necessary and enabled the attainment of positive (PPA) and negative percentage agreement and positive and negative predictive values of 91.4%, 100%, 100%, and 98.3%. Three false negative results remained (two *E. coli* and one *N. meningitidis*). *E. coli* presented the poorest PPA (50%). *Conclusion*: This work confirms the strong performance of the EP, of particular interest in cases of antibiotic therapy before lumbar puncture.

## 1. Introduction

The prognosis of bacterial meningitis remains strongly correlated to the timing of adequate antibiotic treatment initiation [1,2]. Early identification of the etiological infectious agent is helpful to optimize first-line treatments. Therefore, French and European guidelines recommend the use of molecular methods (i) whenever possible when bacterial meningitis is strongly suspected and the direct examination is negative, and (ii) when the direct examination is positive and the culture remains negative at 24 h [3,4]. In that context, multiplex syndromic panel-based molecular approaches present attractive features, including the need for only a small volume of cerebrospinal fluid (CSF), the ease of use, and the low turnaround time [5]. Few molecular multiplex panels have been developed for the diagnosis of bacterial meningitis. Among them, the Eazyplex^®^ CSF direct M panel (AmplexDiagnostics GmbH, Gars-Bahnhof, Germany) can detect the six most common meningeal pathogens thanks to a multiplex real-time Loop-Mediated Isothermal Amplification (LAMP) approach. To date, very little data is available concerning this panel [6]. This study aimed to assess the performance and the accuracy of the Eazyplex^®^ panel (EP) through a retrospective analysis of a routine use of the technique.

## 2. Materials and Methods

### 2.1. Clinical Samples

We retrospectively identified patients with suspected meningitis whose CSF samples were tested with the Eazyplex^®^ CSF direct M panel (AmplexDiagnostics GmbH, Gars-Bahnhof, Germany), at the physician’s and/or microbiologist’s discretion, from December 2016 to December 2019, at Nantes University Hospital. Redundant CSF specimens (i.e., sampled from the same patient during the same clinical event) were excluded. Samples, results, and data were recorded during normal medical care of patients by the professionals who were monitoring them.

### 2.2. Ethics Approval

According to French and European legislation, the use of data in a retrospective monocentric study does not need the approval of the ethics committee. This study was recorded at Nantes hospital by the local Data Privacy Officer under the reference: TS005.BIO.AP.2019_9.

### 2.3. Eazyplex^®^ CSF Direct M Testing

An amount of 125 µl from each sample was subjected to Eazyplex^®^ CSF direct M panel (V1.0) testing according to the manufacturer’s instructions. This panel test consists of a multiplex real-time LAMP assay for the identification of the 6 most common agents of community-acquired and neonatal bacterial meningitis (*Neisseria meningitidis*, *Streptococcus pneumoniae*, *Escherichia coli*, *Streptococcus agalactiae*, *Haemophilus influenzae*, and *Listeria monocytogenes*). The test contains a LAMP inhibition internal control. After a 30 min run, the Eazyplex^®^ software (eazyReportTM, Gars-Bahnhof, Germany) performs automated results analysis according to real-time fluorescence detection of amplification products. If internal control fails, the software automatically provides the result “invalid” for all the panel analyses. In a valid run, each target is reported as “positive” or “negative”. Time to positivity is also mentioned in the report. Although amplification curves and rates analyses are not recommended by the manufacturer, they were systematically analyzed and compared with the Eazyplex^®^ software automated results analysis.

### 2.4. Routine Testing

CSF White Blood Cell (WBC) count and differential (performed if WBC ≥ 10 cells/µL), Red Blood Cell (RBC) count, Gram staining (performed using cytocentrifuged CSF if WBC ≥ 10 cells/µL), and bacterial cultures were performed on every specimen enrolled, as part of the routine care. According to the standard laboratory procedures, the CSF was plated on horse blood and chocolate agar media (bioMérieux, Marcy-l’Étoile, France), inoculated on a Brain Heart Infusion broth (Becton Dickinson, Grenoble, France), and incubated at 37 °C in 5% CO_2_ for 2 to 7 days. Isolated bacteria were identified using VitekMS (bioMérieux, Marcy-l’Étoile, France) matrix-assisted laser desorption ionization–time of flight (MALDI-TOF) mass spectrometry analysis.

### 2.5. Data Analysis and Discrepancy Investigation

Patients’ demographic data, CSF chemistry results (protein and glucose), additional microbiological analysis (specific *N. meningitidis*, *S. pneumoniae* or *L. monocytogenes* PCR, *S. pneumoniae* soluble antigen detection, and 16S rRNA gene PCR and sequencing), PCR testing for virus (enterovirus, herpes simplex virus 1 and 2 (HSV-1/2), and varicella–zoster virus (VZV)), concomitant positive blood culture, antimicrobial therapies administered before lumbar puncture and final clinical diagnosis were retrospectively recorded. When necessary, medical files were n necessary, medical files were retrospectively assessed by infectious diseases specialists blinded to the EP results, to confirm the final diagnosis. All data collected from patients’ medical folders were filled in on a board under an anonymized code.

Positive biological criteria for bacterial meningitis were defined as (i) a positive CSF culture to one of the pathogens included in the EP, and/or (ii) CSF parameters indicative of infection (i.e., the association of WBC count of ≥ 5 cells/µL and protein concentration ≥ 0.5 g/L, and either (i) CSF glucose level ≤ 60% of blood glucose level or CSF glucose level < 2.6 mM (for newborns ≤ 2 months) or (ii) CSF glucose level ≤ 40% of blood glucose level or CSF glucose level < 2.6 mM (for children > 2 months and adults), according to the 2018 French infectious diseases society (French acronym SPILF) recommendations and the 2004 Infectious Diseases Society of America guidelines [3,7]).

A discrepancy investigation was performed for samples with (i) a positive result by the EP, and/or (ii) a concomitant positive blood culture to one of the pathogens included in the EP, and/or (iii) presenting positive biological criteria for bacterial meningitis (see above). Molecular test results were analyzed according to blood and CSF cultures, CSF chemistry parameters as well as previous antibiotics administration and final clinical diagnosis. After resolution of discordant results, the EP results were considered true positive (TP), true negative (TN), false positive (FP), or false negative (FN). If the available data did not support nor exclude a diagnosis of meningitis due to one of the six pathogens targeted, the result was considered inconclusive.

### 2.6. Eazyplex^®^ Panel Performance Analysis

Positive Percentage Agreement (PPA), Negative Percentage Agreement (NPA), Positive Predictive Value (PPV), and Negative Predictive Value (NPV) were calculated as 100*[TP/(TP + FN)], 100*[TN/(TN + FP)], 100*[TP/(TP + FP)], and [TN/(TN + FN)] respectively. The terms “PPA” and “NPA” were used instead of “sensitivity” and “specificity” because a “non-gold standard” assay was used for the comparator analysis.

### 2.7. Statistical Analysis

CSF protein concentration, WBC and RBC counts were compared between the CSF samples with successful or failed internal inhibition control using an unpaired *t* test (GraphPad prism (La Jolla, CA, United States)). *p* < 0.05 was conventionally used for statistical significance.

## 3. Results

### 3.1. Population and Sample Description

**Figure 1:**
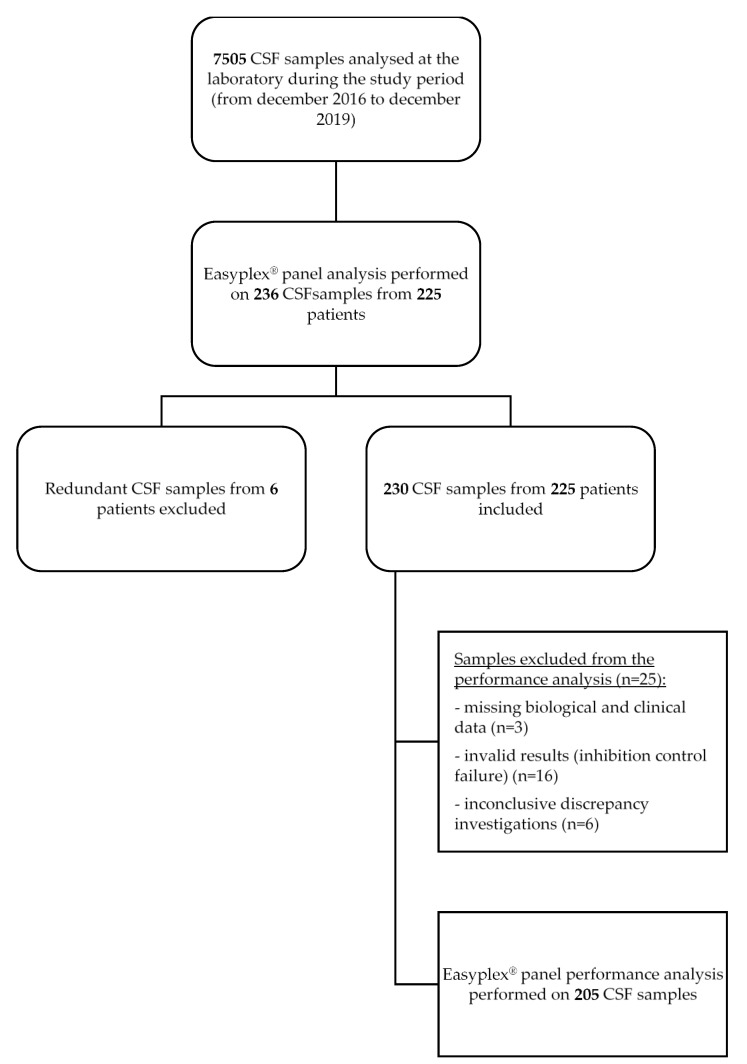
Study selection flow diagram. Abbreviations: CSF, cerebrospinal fluid; LAMP, Loop-mediated isothermal amplification.

During the study period, 7505 CSF specimens were analyzed, of which 236 samples from 225 patients were subjected to EP testing. Redundant samples from six patients were excluded. Thus, a total of 230 CSF samples from 225 patients were included in the analysis (Figure 1). Patients were 44% female (*n* = 101) and 56% male (*n* = 129). The age distribution included 171 (74%) adults 16 years of age or older and 59 (26%) pediatric patients distributed as followed: 24 infants of ≤ 2 months of age, six between 3 and 11 months old, and 29 children between 1 and 15 years old. CSF specimens were sampled in patients from the emergency care units (*n* = 85), intensive care units (*n* = 52), medical care units (*n* = 59), or from neighborhood hospitals (*n* = 34).

### 3.2. Impact of CSF Protein Concentration, WBC and RBC Counts on LAMP Inhibition

**Figure 2:**
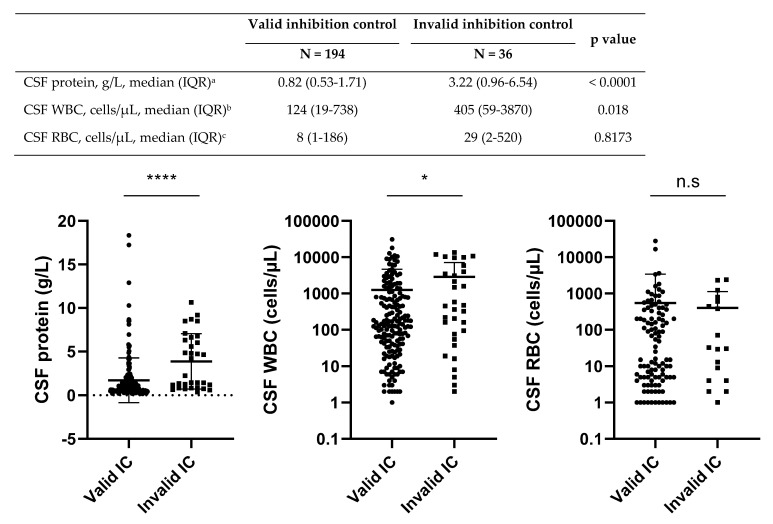
Impact of CSF protein concentration, WBC and RBC count on LAMP inhibition. Abbreviations: CSF, cerebrospinal fluid; IQR, interquartile range; WBC, white blood cell; RBC, red blood cell; IC, inhibition control. ^a^ missing data: valid IC group (*n* = 9) and invalid IC group (*n* = 4); ^b^ missing data: valid IC group (*n* = 10) and invalid IC group (*n* = 4); ^c^ missing data: valid IC group (*n* = 63) and invalid IC group (*n* = 15). * *p* < 0.05; **** *p* < 0.0001; n.s: not significant.

The internal control failed on the first attempt for 36 of the 230 CSF tested, suggesting a LAMP inhibition (inhibition rate of 15.6%). The median (interquartile range (IQR)) CSF protein concentration was 0.82 g/L (0.53–1.71) in CSF with valid inhibition control versus 3.22 g/L (0.96–6.54) in CSF with invalid inhibition control. The median (IQR) WBC count was 124 cells/µL (19–738) in CSF with valid inhibition control versus 405 cells/µL (59–3870) in CSF with invalid inhibition control. The median (IQR) RBC count was 8 cells/µL (1–186) in CSF with valid inhibition control versus 29 cells/µL (2–520) in CSF with invalid inhibition control. Statistical analysis revealed CSF protein concentration and WBC count were significantly higher in CSF with invalid inhibition control (Figure 2). Over the 36 samples unsuccessfully tested in the first attempt, 17 were centrifuged (4000 RPM, 1 min) and subjected to a new Eazyplex^®^ analysis. Inhibition control came back successful in 15 out of the 17 reanalyzed CSF samples. Nineteen CSF were not reanalyzed: five of them presented a positive target, and 14 an insufficient volume.

### 3.3. Amplification Curves Analysis

**Figure 3:**
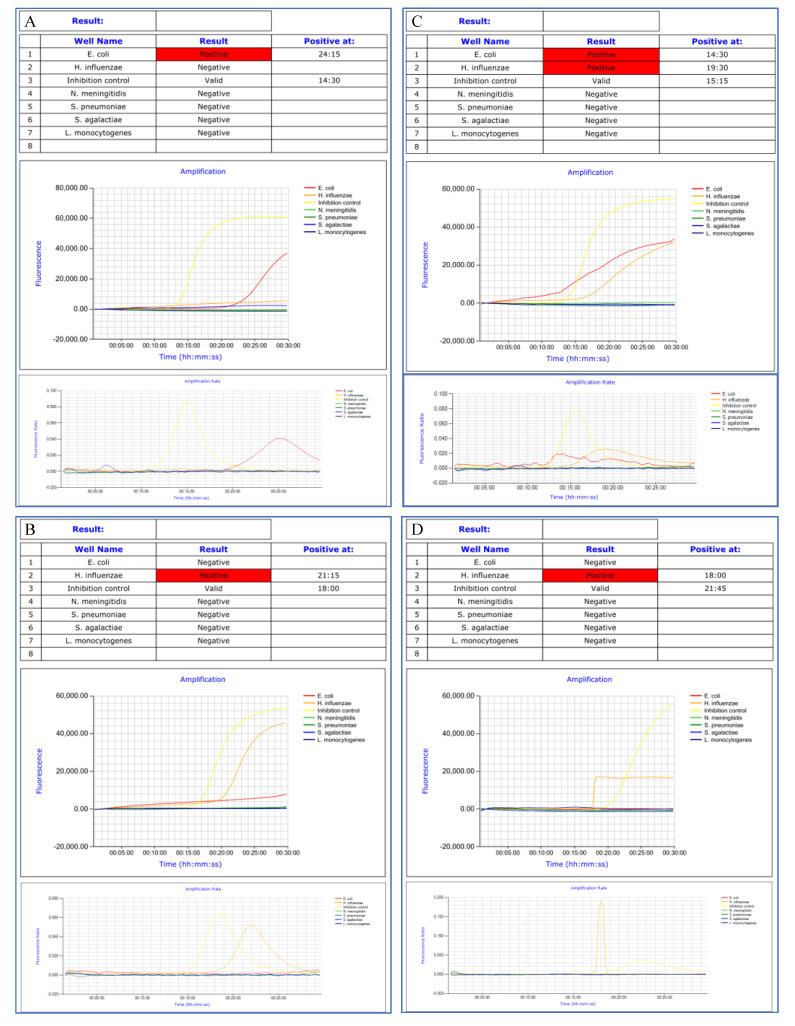
Amplification curves and rates of true positive (**A**,**B**) and false positive (**C**,**D**) results.

**Figure 4:**
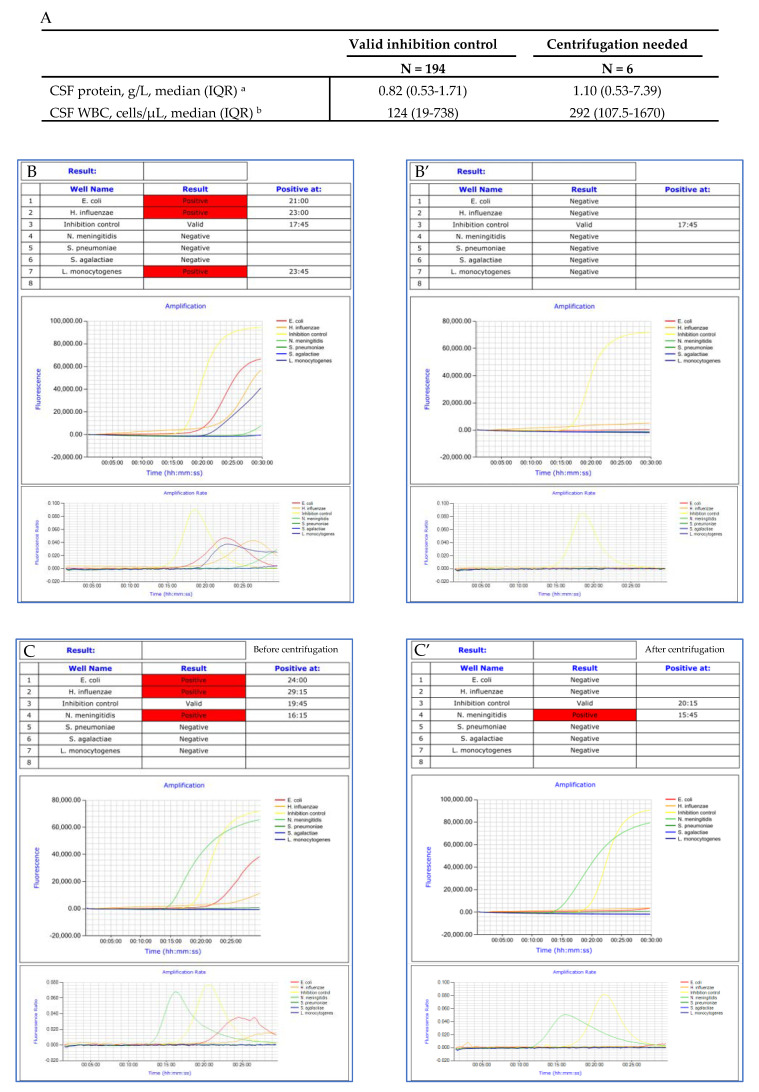
Impact of CSF centrifugation on amplification curves. CSF protein and WBC in valid inhibition control group versus in CSF needed centrifugation before analysis (**A**); Amplification curves and rates before (**B**,**C**) and after (**B’**,**C’**) centrifugation. Abbreviations: CSF, cerebrospinal fluid; WBC, white blood cells. ^a^ missing data: valid IC group (*n* = 9) and centrifugation needed group (*n* = 1); ^b^ missing data: valid IC group (*n* = 10) and invalid IC group (*n*=1).

The software was proved deficient in 13 cases, yielding false positive results for *H. influenzae* (*n* = 8), *E. coli* (*n* = 4), and *L. monocytogenes* (*n* = 1). Biological expertise of the amplification curves and rates concluded that the observed amplifications were non-specific (Examples of amplification curves of true and false positive results are presented in Figure 3). Time to positivity was more than 25 min in 11 out of the 13 cases. Six samples presenting non-specific amplification curves were centrifuged (4000 RPM, 1 min) and reanalyzed. Centrifugation facilitated a reduction in non-specific reactions (Figure 4).

Amplification curve analysis also allowed the rectification of five false negative results (*N. meningitidis* (*n* = 3) and *S. pneumoniae* (*n* = 2)), not mentioned by the software because internal control failed. In such situations, the software automatically provided the result “invalid” for all the panel analyses, irrespective of other target results.

### 3.4. Eazyplex^®^ Panel Results

Overall, after biological analysis and exclusion of the false positive and negative results generated by the software, the EP testing yielded positive, negative, and invalid results for 32 (13.9%), 182 (79.1%), and 16 (7%) CSF samples, respectively. The highest detection rates were in the pediatric age groups. The relative prevalence of each pathogen among the positive samples is presented in Table 1. The most prevalent organisms detected during this study were *N. meningitidis* (*n* = 16) and *S. pneumoniae* (*n* = 9). Gram stain was negative for 11 (34%) of the 32 EP positive samples. Cultures remained negative for 14 (44%) of the 32 EP positive samples: antimicrobial therapies were administered before lumbar puncture in 11 cases, and the last three cases were meningococcal meningitis.

### 3.5. Eazyplex^®^ Panel Performance

Nineteen samples were excluded from the performance analysis: three due to missing biological and clinical data, and 16 to invalid results (inhibition control failures). Discrepancy investigations remained inconclusive for six samples. Overall, EP performance was evaluated on 205 CSF analysis (Figure 1). Discrepancy investigation identified three FN results: the EP missed the identification of two *E. coli* meningitis and one *N. meningitidis* meningitis. Internal inhibition control was valid in the three cases. No FP results were observed (results of the discrepancy investigation are presented in Table S1 in the supplementary material). PPA, NPA, PPV, and NPV were calculated with respect to the conclusions of the discrepancy investigation. Overall, the EP demonstrated PPA, NPA, PPV, and NPV of 91.4%, 100%, 100%, and 98.3%, respectively. The performance characteristics for individual EP targets are presented in Table 2. The poorest positive percentage of agreement was reported with *E. coli* (PPA = 50%).

## 4. Discussion

Syndromic multiplex molecular tests are promising tools for the rapid etiological diagnosis of bacterial meningitis. Accuracy and impact on the patients’ care of the FilmArray^®^ meningitis/encephalitis (FA-ME) panel have been assessed several times [8,9,10,11,12]. The Eazyplex^®^ CSF direct M panel assay, a multiplex real-time LAMP assay, provides another alternative for the rapid diagnosis of bacterial meningitis. However, clinical validation data of this panel are still scarce, as only one study has assessed its performance, without conducting any discrepant analysis [6].

We reported here the strong performance of the EP in meningeal pathogen detection (PPA, NPA, PPV, and NPV of 91.4%, 100%, 100%, and 98.3%, respectively), comparable to that reported in the only other study already published (sensitivity 90.9%, specificity 100%, NPV 100%, NPV 95.8%) [6]. These performances were also similar to those demonstrated by the FA-ME panel in a recent meta-analysis, with summary sensitivity and specificity estimated at 90% and 97%, respectively [13].

Among 32 patients with meningitis, in 14 cases microbiological documentation was only based on the EP results while cultures remained negative. The EP was of particular interest in cases of antibiotic intake before lumbar puncture (*n* = 11), or in meningococcal meningitis (*n* = 3). These conclusions were consistent with the literature since (i) antibiotic treatments before lumbar puncture decreased the yield of CSF culture by 10 to 20%, and (ii) CSF positivity differed with causative microorganisms, with the lowest positivity rates in meningococcal meningitis [14,15]. However, one case of meningococcal meningitis with antimicrobial agents before lumbar punction was not identified through EP, whereas blood culture was positive.

Our work described invalid results due to LAMP inhibition for 7% of the CSF samples analyzed, and underlined the impact of CSF protein concentration and WBC count on LAMP inhibition. Sample centrifugation before analysis significantly reduced interference (from 15.6% to 7% of the analyzed CSF specimens). We thus suggest centrifugation should be performed before analysis whenever chemistry and cytological parameters are high.

Furthermore, the low PPA obtained with *E. coli* suggests a limitation of the panel regarding this species, and indicates the need for cautious interpretation of negative results in cases of clinically suspected *E. coli* meningitis. Over 135 CSF samples analyzed, D’Inzeo et al. did not mention any FN results generated by the EP. They reported only four cases due to microorganisms not included in the panel [6]. However, no discrepancy analysis was performed and 16S rRNA PCR was considered as the gold standard. According to the literature, poor reported sensitivity of 16S rRNA PCR for bacterial meningitis diagnosis makes its use as a gold standard questionable [16]. Regarding *E. coli* detection, the literature on the FA-ME did not underscore lower PPA; only one study reported one FN result [12].

Our work also highlighted the need for systematic biological expertise of the amplification curves to avoid false positive and negative results generated by the software analysis. According to a systematic review conducted on the accuracy of the FA-ME panel, 4% (32/807) of the positive results generated by the FA-ME panel were considered FP results after discrepant analysis [13]. The highest proportion of FP results was observed for *S. pneumoniae* (17.5%) followed by *S. agalactiae* (15.4%). *S. pneumoniae* FP results were mostly attributed to samples or laboratory contaminations. No mention was made of the amplification curve analysis. The availability of the amplification curves and rates appeared as a significant added value of the Eazyplex^®^ technology and we strongly recommend their systematic analysis, including when internal control fails.

This work was the largest evaluation of the EP performance to date, and relies on a well-conducted discrepancy analysis, including retrospective assessment of medical files by infectious diseases specialists. However, some limitations should be acknowledged. Firstly, this work was limited by the low percentage of positive results. Secondly, invalid results were excluded from the performance assessment, which could lead to an overestimation of these performances. Thirdly, this work was performed with the first version (V1.0) of the Eazyplex^®^ CSF direct M panel. The manufacturer has recently marketed a new version of this test (V1.1), notably including an additional reagent to reduce the inhibitory influence of CSF components. Moreover, the new manufacturer’s instructions recommend confirming positive reactions with another technology when the time to positivity is greater than 25 min. This recommendation would have reduced the FP results generated by the automated software.

When compared with the FA-ME panel (designed to identify 14 targets, including seven viruses and *Cryptococcus neoformans/gattii*), the EP detects fewer targets but offers the benefits of a focus on bacterial meningitis diagnosis and a lower price [5]. Furthermore, the LAMP technology is easier to perform and only requires a heat block or water bath for amplification [17]. For these reasons, the EP has the potential to make the urgently needed diagnosis for bacterial meningitis affordable for resource-limited settings of both developed and developing countries [5,18].

## 5. Conclusions

This large retrospective evaluation of the EP in the diagnosis of bacterial meningitis (i) confirmed the strong performance of the panel, of particular interest in cases of antibiotic intake before lumbar puncture, (ii) underlined the significant impact of CSF protein concentration and WBC counts in LAMP inhibition, (iii) demonstrated the need for systematic biological expertise of the amplification curves, and (iv) observed the limitation of the panel for *E. coli* detection. Multiplex syndromic panels are easy to use and valuable additional tests to improve the timeliness of pathogen identification. However, this work underscores the fact that informed expertise on such automated approaches remains essential. 

## Figures and Tables

**Table 1:** Easyplex^®^ panel results for all samples and by age groups.

	All Samples	Results by Age Groups				
	N = 230	≤2 Months	3–11 Months	1–15 Years	16–64 Years	≥65 Years
		N = 24	N = 6	N = 29	N = 117	N = 54
Positive results, number (% of total)	32 (13.9)	3 (12.5)	3 (50)	8 (27.6)	14 (12)	4 (7.4)
*N. meningitidis*, number (% of positive samples)	16 (50)	0 (0)	2 (66.7)	5 (62.5)	8 (57.2)	1 (25)
*S. pneumoniae*, number (% of positive samples)	9 (28)	0 (0)	1 (33.3)	2 (25)	3 (21.5)	3 (75)
*E. coli*, number (% of positive samples)	2 (6.3)	1 (33.3)	0 (0)	0 (0)	1 (7.1)	0 (0)
*S. agalactiae*, number (% of positive samples)	2 (6.3)	1 (33.3)	0 (0)	0 (0)	1 (7.1)	0 (0)
*H. influenzae*, number (% of positive samples)	2 (6.3)	0 (0)	0 (0)	1 (12.5)	1 (7.1)	0 (0)
*L. monocytogenes*, number (% of positive samples)	1 (3.1)	1 (33.3)	0 (0)	0 (0)	0 (0)	0 (0)
Negative results, number (% of total)	182 (79.1)	18 (75)	3 (50)	20 (69)	93 (79.5)	48 (88.9)
Invalid results, number (% of total)	16 (7)	3 (12.5)	0 (0)	1 (3.4)	10 (8.5)	2 (3.7)

**Table 2:** Performance of the Easyplex^®^ panel overall and for individual targets.

Target	PPA		NPA		PPV		NPV	
	TP/(TP + FN)	%	TN/(TN + FP)	%	TP/(TP + FP)	%	TN/(TN + FN)	%
*N. meningitidis*	16/17	94.1	170/170	100	16/16	100	170/171	99.4
*S. pneumoniae*	9/9	100	170/170	100	9/9	100	170/170	100
*E. coli*	2/4	50	170/170	100	2/2	100	170/172	98.8
*S. agalactiae*	2/2	100	170/170	100	2/2	100	170/170	100
*H. influenzae*	2/2	100	170/170	100	2/2	100	170/170	100
*L. monocytogenes*	1/1	100	170/170	100	1/1	100	170/170	100
Overall	32/35	91.4	170/170	100	32/32	100	170/173	98.3

## Supplementary Materials

The following are available online at https://www.mdpi.com/article/10.3390/microorganisms9091859/s1, Table S1: Discrepancy investigation.
