# Supplementary material for: Assessment of a Multiplex LAMP Assay (Eazyplex® CSF Direct M) for Rapid Molecular Diagnosis of Bacterial Meningitis: Accuracy and Pitfalls
Source: Microorganisms. 2021 Sep 1;9(9):1859. doi: 10.3390/microorganisms9091859 (PMC8471247; doi:10.3390/microorganisms9091859)
Supplement: Supplementary file 1 [file microorganisms-09-01859-s001.zip › microorganisms-1327353-supplementary.pdf]

			CSF parameters								Discrepancy investigation				
Patient N°	Target	Age (years)[a], sex	WBC (cells/µL)	Neutrophils (%)	RBC (cells/µL)	Protein (g/L)	Glucose (mmol/L)	Ratio of CSF to serum glucose	Gram stain	Bacterial culture	Antimicrobial agents given before LP	Positive blood culture to the target	Additional analysis	Final clinical diagnosis	Final resolution of discrepancy
FALSE POSITIVE ANALYSIS															
1	*N. meningitidis*	14,M	6500	90	200	8,11	1,40	0,11	GNDC	STERILE	No	No	Specific *N. meningitidis* PCR +	Meningococcal meningitis	TP
2	*S. pneumoniae*	23,M	672	90	RR	MD	MD	MD	GPDC	*S. pneumoniae*	Yes	No		Pneumococcal otitis and meningitis	TP
3	*N. meningitidis*	4m,F	3860	80	RR	2,33	<0,10	0,02	GNDC	*N. meningitidis*	No	Yes		Meningococcal meningitis	TP
4	*S. pneumoniae*	45,F	3120	95	QQ	6,31	<0,10	0,01	GPDC	STERILE	Yes	Yes	*S. pneumoniae* soluble antigen detection	Pneumococcal meningitis	TP
5	*E. coli*	11d,F	3500	85	RR	2,05	2,10	MD	NOS	STERILE	Yes	Yes		Maternal-neonatal meningitis	TP
6	*N. meningitidis*	22,F	7600	98	200	5,66	0,40	0,03	GNDC	*N. meningitidis*	MD	No	Specific *N. meningitidis* PCR +	Meningococcal meningitis	TP
7	*N. meningitidis*	10,F	9920	98	800	6,62	<0,10	0,01	GNDC	STERILE	Yes	No	Specific *N. meningitidis* PCR +	Meningococcal meningitis	TP
8	*H. influenzae*	39,M	18000	85	1300	10,28	<0,1	0,01	GNB	*H. influenzae*	MD	Yes		*H. influenzae* meningitis	TP
9	*N. meningitidis*	11,M	3200	MD	QQ	4,87	1,70	0,30	GNDC	STERILE	Yes	No	Specific *N. meningitidis* PCR +	Meningococcal meningitis	TP
10	*S. pneumoniae*	3m,M	440	50	15	1,38	<0,10	0,01	GPDC	*S. pneumoniae*	No	Yes		Pneumococcal meningitis	TP
11	*N. meningitidis*	27,M	6080	95	RR	1,69	2,80	0,42	GNDC	*N. meningitidis*	No	No blood culture	Specific *N. meningitidis* PCR +	Meningococcal meningitis	TP
12	*N. meningitidis*	29,M	3000	67	MD	0,48	MD	MD	NOS	MD	Yes	Yes	Specific *N. meningitidis* PCR +	Meningococcal meningitis	TP
13	*H. influenzae*	1,M	292	50	12	0,70	3,40	MD	NOS	*H. influenzae*	Yes	No		*H. influenzae* meningitis	TP
14	*L. monocytogenes*	1d,M	TI	QQ	NB	8,52	1,30	0,19	NOS	STERILE	Yes	No		Maternal-neonatal meningitis	TP
15	*S. agalactiae*	40,F	1920	81	AN	3,65	<0,10	0,01	GPDC	*S. agalactiae*	No	Yes		Post-operative meningitis	TP
16	*N. meningitidis*	20,M	10000	75	200	3,13	4,60	0,60	GNDC	*N. meningitidis*	No	No		Meningococcal meningitis	TP
17	*S. pneumoniae*	2,M	4360	80	400	1,38	1,30	MD	NOS	*S. pneumoniae*	Yes	Yes		Pneumococcal meningitis	TP
18	*N. meningitidis*	72,F	4640	98	RR	5,80	0,60	MD	GNDC	*N. meningitidis*	No	Yes	Specific *N. meningitidis* PCR +	Meningococcal meningitis	TP
19	*S. pneumoniae*	68,M	>12000	90	NB	8,49	<0,10	MD	GPDC	*S. pneumoniae*	Yes	No	*S. pneumoniae* soluble antigen detection	Pneumococcal meningitis	TP
20	*E. coli*	20,M	770	95	AN	12,90	<0,10	MD	GNB	*E. coli*	Yes	No		Post-operative meningitis	TP
21	*N. meningitidis*	4m,M	3400	80	560	5,06	0,80	0,10	GNDC	*N. meningitidis*	No	No	Specific *N. meningitidis* PCR +	Meningococcal meningitis	TP
22	*S. pneumoniae*	66,F	5920	90	600	7,08	5,80	0,35	NOS	STERILE	Yes	Yes		Pneumococcal meningitis	TP
23	*N. meningitidis*	48,M	5	MD	28000	2,40	11,60	2,96	NOS	STERILE	Yes	No	Specific *N. meningitidis* PCR + (serum)	Meningococcal severe sepsis leading to death	TP
24	*S. pneumoniae*	72,F	1600	95	500	8,52	<0,10	MD	GPDC	STERILE	No	Yes		Pneumococcal meningitis	TP
25	*N. meningitidis*	30,M	9760	86	2320	8,48	0,10	0,01	NOS	*N. meningitidis*	No	No	Specific *N. meningitidis* PCR +	Meningococcal meningitis	TP
26	*S. pneumoniae*	3,M	124	90	354	2,94	<0,10	0,01	GPDC	*S. pneumoniae*	No	Yes		Pneumococcal meningitis	TP
27	*S. pneumoniae*	30,F	4000	95	380	6,77	0,23	MD	NOS	*S. pneumoniae*	No	No blood culture	Specific S. *pneumoniae* PCR +	Pneumococcal meningitis	TP
28	*N. meningitidis*	3,M	1380	95	80	MD	MD	MD	NOS	STERILE	Yes	No	Specific *N. meningitidis* PCR +	Meningococcal meningitis	TP
29	*N. meningitidis*	18,F	2263	82	143	1,42	2,18	0,34	NOS	STERILE	Yes	No	Specific *N. meningitidis* PCR +	Meningococcal meningitis and purpura	TP
30	*N. meningitidis*	2,M	8800	90	160	1,48	0,90	MD	GNDC	STERILE	Yes	No	Specific *N. meningitidis* PCR +	Meningococcal meningitis	TP
31	*S. agalactiae*	14d,F	185	100	NB	2,15	0,70	MD	GPC	*S. agalactiae*	No	Yes		Maternal-neonatal meningitis	TP
32	*N. meningitidis*	23,F	3480	92	440	5,58	0,10	0,01	GNDC	STERILE	No	No	Specific *N. meningitidis* PCR +	Meningococcal meningitis	TP
FALSE NEGATIVE ANALYSIS															
33	*E. coli*	8d,F	TI	RR	AN	1,93	1,70	MD	NOS	*E. coli*	Yes	Yes		Maternal-neonatal meningitis	FN
34	*E. coli*	71,F	3600	95	660	17,24	5,50	0,63	NOS	STERILE	Yes	Yes	Negative 16S rRNA gene PCR	*E. coli* meningitis presumed	FN
35	*N. meningitidis*	20,M	2	NP	15	0,28	4,00	0,66	NP	STERILE	No	Yes		Bacteremia without meningitis	TN
36	*N. meningitidis*	2,M	113	95	186	0,45	5,50	MD	NOS	STERILE	Yes	Yes		Purpura and presumed meningitis	FN
37	*S. pneumoniae*	20,M	4	NP	MD	0,32	10,30	1,51	NP	STERILE	Yes	Yes		Pneumonia	TN
38	*S. pneumoniae*	46,M	7	NP	8	0,29	4,20	MD	NOS	STERILE	No	Yes		Infectious endocarditis	TN
39	*E. coli*	2d,F	4	NP	182	1,22	4,50	MD	NP	STERILE	Yes	Yes		Severe sepsis without meningitis	TN
40	*S. agalactiae*	16d,M	1	NP	1	0,61	2,60	MD	NOS	STERILE	No	Yes		Osteomyelitis	TN
41	*S. agalactiae*	5d,F	2	NP	1	1,44	3,10	MD	NOS	STERILE	Yes	Yes		Chorioamnionitis and maternal-neonatal infection without meningitis	TN
42	*N. meningitidis*	50,F	<1	NP	4	0,20	4,10	MD	NP	STERILE	Yes	Yes		Bacteremia without meningitis	TN
43		14d,M	1920	50	5	1,22	2,80	0,49	NOS	STERILE	No	No	EV PCR +	Enterovirus meningitis	TN
44		13d,M	1500	70	16600	1,02	2,20	MD	NOS	STERILE	Yes	No		Rotavirus enteritis	TN
45		18d,F	34	MD	5	0,73	3,80	0,44	NOS	STERILE	Yes	No		Sepsis without documentation	TN
46		8d,M	2720	12	<1	1,75	2,20	0,37	NOS	STERILE	No	No	EV PCR +	Enterovirus meningitis	TN
47		1d,M	800	5	QQ	1,34	0,90	MD	NOS	STERILE	Yes	No		Severe sepsis leading to death, mother suffering from a *E. coli* urinary tract infection	TN
48	*S. agalactiae*	1m,M	IQ	IQ	IQ	1,42	2,60	MD	NOS	STERILE	No	Yes		Bacteremia without meningitis	TN
49		9d,M	454	26	MD	3,71	3,00	0,56	NOS	STERILE	No	No blood culture		Epilepsy	TN
50		20d,M	453	1	<1	2,00	0,97	0,19	NOS	STERILE	No	No	EV PCR +	Enterovirus meningitis	TN
51		27,F	82	70	MD	3,40	0,48	0,09	NOS	STERILE	No	No	EV PCR +	Enterovirus meningitis	TN
52		68,M	250	95	NB	8,67	2,30	0,22	NOS	STERILE	Yes	No	*Strepctococcus mitis/oralis* + blood culture	Meningitis and severe sepsis in a cirrhotic patient	TN
53		67,F	8400	92	200	3,28	0,10	0,01	NOS	*S. intermedius*	No	No	*Parvimonas micra* + blood culture	Brain abscess due to *Streprococus intermedius*	TN
54		74,F	13600	98	32	6,02	1,60	0,10	NOS	STERILE	No	No	Negative 16S rRNA gene PCR	Brain abscess and ventriculitis without documentation	TN
55		64,F	130	60	90	0,80	1,80	0,29	NOS	STERILE	No	No		Meningoencephalitis without documentation	TN
56		13,M	660	1	<1	1,74	2,00	0,39	NOS	STERILE	No	No		Meningoencephalitis without documentation, the outcome was quickly favorable (48H)	TN
57		39,F	840	90	QQ	0,86	2,00	0,39	NOS	STERILE	No	No	EV PCR +	Enterovirus meningitis	TN
58		63,M	783	16	130	2,23	3,80	0,40	NOS	STERILE	Yes	No	VZV PCR +	VZV encephalitis	TN
59		9,M	26	15	1	4,17	1,90	0,29	NOS	STERILE	No	No		Neoplastic meningitis	TN
60		74,M	217	80	29	4,79	0,70	0,08	NOS	STERILE	Yes	No	*Streptococcus dysgalactiae* + blood culture	Severe sepsis with neurological disorders	TN
61		47,M	180	90	<1	1,73	4,20	0,28	NOS	*C. albicans*	No	No		Candida meningiradiculitis	TN
62		11,F	125	41	RR	0,64	3,30	0,35	NOS	STERILE	Yes	No	Negative 16S rRNA gene PCR	Maxillary sinusitis and empyema	TN
63		55,M	200	80	1120	1,17	1,60	0,25	NOS	STERILE	No	No		Non-infectious post-operative (lumpectomy) meningitis	TN
64		52,M	10800	80	1240	8,70	1,20	0,14	NOS	STERILE	No	No		Brain abscess due to *S. intermedius*	TN
65		56,M	12800	90	RR	4,36	2,10	MD	NOS	STERILE	No	No		Non-infectious meningitis (glioblastoma)	TN
66		35,M	1400	50	<1	1,49	2,30	MD	NOS	STERILE	Yes	No	Negative specific *S. pneumoniae*, *N. meningitidis, HSV and EV* PCR and negative 16S rRNA gene PCR	Meningitis without documentation (ventriculoperitoneal shunt)	TN
67		36,F	140	0	NB	2,97	2,60	MD	NOS	STERILE	No	No		Lymphoma	TN
68		71,M	216	70	AN	1,35	1,80	MD	NOS	STERILE	No	No	*S. mitis/oralis* + blood culture	Severe sepsis with neurological disorders	TN
69		52,M	10500	95	500	3,61	0,70	MD	NOS	STERILE	No	No	*Klebsiella oxytoca*, *Streptococcus anginosus* and *Bacteroides fragilis* isolated in a subsequent lumbar punction (2 days after)	Stercoral meningitis (shunt migration in ventriculoperitoneal shunting)	TN
70		62,F	18	MD	290	0,82	2,10	MD	NOS	STERILE	No	No	EV PCR +	Enterovirus meningitis	TN
71		56,M	350	25	<1	1,07	9,60	MD	NOS	STERILE	Yes	No		Lymphocytic meningoencephalitis without documentation	TN
72		24,F	2920	75	RR	1,30	1,40	MD	NOS	STERILE	No	No	Negative 16S rRNA gene PCR	Post-operative (lumpectomy) meningitis without documentation	TN
73		33,F	95	0	6	0,60	MD	MD	NOS	STERILE	No	No		Multiple sclerosis	TN
74		65,F	58	26	34	0,88	1,50	MD	NOS	STERILE	No	No		Neurogical maninfestations of a systemic lupus erythematosus	TN
75		78,M	41	0	12	2,09	0,30	MD	NOS	STERILE	MD	No		Neoplastic meningitis	TN
76		51,F	160	80	NB	2,00	0,60	MD	NOS	STERILE	No	No	Negative 16S rRNA gene PCR	Subarachnoid hemorrhage	TN
77		30,F	127	35	3	MD	MD	MD	NOS	STERILE	No	No blood culture		Rhinitis	TN
78		70,F	1100	85	RR	1,33	2,40	MD	NOS	STERILE	No	No		Brain tumor	TN
79		78,M	66	MD	<1	MD	MD	MD	NOS	STERILE	Yes	No		Lymphocytic meningoencephalitis without documentation	TN
80		22,F	560	80	593	1,30	2,10	MD	NOS	STERILE	No	No		Post-operative meningitis without documentation	TN
81		21,M	11200	95	RR	3,69	<0,10	MD	GPDC	*S. epidermidis*	No	No		EVD associated meningitis	TN
82		57,M	203	0	QQ	MD	MD	MD	NOS	STERILE	No	No	High HIV viral load in CSF	HIV encephalitis	TN
83		62,M	57	95	<1	1,04	2,40	MD	NOS	STERILE	No	No		Brain hematoma following a traumatic brain injury	TN
84		70,M	700	85	MD	1,13	1,70	MD	NOS	STERILE	Yes	No		Clinical recurrence (fever and headache) of pneumococal meningitis after 17 days of treatment	TN
85		78,F	1035	55	200	2,32	2,50	MD	NOS	STERILE	No	No	Negative 16S rRNA gene PCR	Post-operative (enucleation) meningitis without documentation	TN
86	*S. agalactiae*	23d,M	11	MD	68	0,75	1,40	0,20	NOS	STERILE	Yes	Yes		Maternal-neonatal infection and possible meningitis	inconclusive
87		6,M	732	91	MD	6,95	1,10	0,10	NOS	STERILE	No	No	Negative 16S rRNA gene PCR	Otitis and severe intracranial hypertension leading to death	inconclusive
88		68,F	31000	97	MD	4,17	2,10	0,19	NOS	STERILE	Yes	No	*S. pneumoniae* soluble antigen negative	Sinusitis and purulent meningitis without documentation (underlying breach)	inconclusive
89		35,M	1920	95	3360	2,11	0,90	0,19	NOS	STERILE	No	No		Suspected bacterial meningitis following a traumatic brain injury	inconclusive
90		68,M	320	85	2400	4,20	1,30	MD	NOS	STERILE	No	No		Meningitis without documentation (underlying breach)	inconclusive
91		23,F	2570	82	QQ	1,87	1,30	MD	NOS	STERILE	No	No	Negative 16S rRNA gene PCR	Meningitis without documentation (medical history of breach)	inconclusive

Table S1. Discrepancy Investigation

a : years unless precised (d, days ; m, months)

Abbreviations: CSF, Cerebral Spinal Fluid ; EV, Enterovirus; GNB, Gram Negative Bacilli; GNDC, Gram Negative DiploCocci ; GPC, Gram Positive Cocci ; GPDC, Gram Positive DiploCocci; HIV, Human Immunodeficiency Virus; IQ, Insufficient Quantity ; LP, Lumbar Punction ; MD, Missing Data ; NOS, No Organisms Seen in Smear ; NP, Not Performed ; FN, False Negative ; FP, False Positive ; RBC, Red Blood Cells ;
TI, Technical Impossibility ; TN, True Negative ; TP, True Positive ; VZV, Varicella Zoster Virus; WBC, White Blood Cells
